# Impact of COVID-19 Pandemic–Induced Changes in Clinical Practicums on the Mental Health of Newly Graduated Nurses: Longitudinal Study

**DOI:** 10.2196/79556

**Published:** 2025-12-23

**Authors:** Takashi Ohue, Yuka Ohue

**Affiliations:** 1 Graduate School of Health Sciences and Faculty of Health Sciences, Okayama University, 2-5-1, Shikata-Cho, Kitaku, Okayama, 700-8558, Japan, 81 0862356841; 2 Department of Nursing, Faculty of Nursing, Hyogo University, Kakogawa, Japan

**Keywords:** nurses, COVID-19, stress, burnout, intention to leave, longitudinal study

## Abstract

**Background:**

The COVID-19 pandemic disrupted nursing education worldwide, particularly clinical practicums, reducing opportunities for hands-on learning. Newly graduated nurses have reported increased stress, reduced confidence, and a higher risk of burnout. However, few studies have examined the long-term mental health effects of these disruptions.

**Objective:**

This study aimed to longitudinally examine how changes in clinical practicums during the COVID-19 pandemic affected the mental health of nurses who graduated in the academic year 2021-2022.

**Methods:**

A quantitative longitudinal study was conducted at 3 time points: June 2022, September 2022, and December 2022. This study assessed the nurses’ demographic data and the perceived impact on the students of disruptions in domain-specific and integration practicums, practicum formats, and clinical difficulty. Instruments used included the Nursing Job Stressor Scale, the Maslach Burnout Inventory, and adapted items for measuring intention to leave the profession. Participants were categorized into high- and low-impact groups. A 2-way ANOVA was used to examine mental health indicators over time.

**Results:**

Participants who perceived a greater impact of practicum disruptions reported substantially higher levels of clinical difficulty and stressors. In the September 2022 survey, those perceiving less impact from the integration practicum reported a stronger intention to continue nursing. In the December 2022 survey, emotional exhaustion, a core component of burnout, was significantly higher in the high-impact group.

**Conclusions:**

The perceived quality and extent of clinical practicum experiences significantly influenced the psychological burden and career intentions of newly graduated nurses. Disruptions caused by the COVID-19 pandemic may have lasting effects on nurses’ mental health. These findings underscore the need for continuous workplace support and targeted mental health interventions for early-career nurses to ensure safe and sustainable nursing practice.

## Introduction

### Background

In December 2019, the first case of COVID-19 was reported in Wuhan, China. The World Health Organization subsequently declared the outbreak a public health emergency of international concern [1]. The global spread of COVID-19 had a profound impact on society as a whole. In particular, the health care sector worldwide experienced significant changes, such as the restructuring of care systems, strengthening of infection control, and resource shortages. Nursing education, which accords immense importance to experiential learning, was also significantly affected. In many countries and regions, hospitals suspended or restricted clinical practicums for nursing students, pivoting toward online training [2,3]. Nursing students indicated that the COVID-19 pandemic significantly increased their stress levels, exacerbating their psychological burden [4].

In Japan, many hospitals ceased accepting students to avoid the spread of infection, resulting in a substantial reduction in traditional clinical practicums at nursing universities. Instead, they introduced on-campus simulations and exercises [5,6]. While these changes were necessary for public health reasons, they raised several challenges from the perspective of fostering practical nursing competencies.

The psychosocial aspects of the COVID-19 pandemic, such as restrictions on practicum opportunities, are also believed to have adversely affected nursing students’ mental health [7]. Clinical practicums aim to integrate and enhance nursing knowledge, skills, and attitudes, cultivating the ability to provide appropriate care based on accurate situational judgment [8]. Therefore, limitations on such experiences may severely hinder the development of these competencies.

Indeed, comparative research on students whose practicum formats were altered because of the COVID-19 pandemic indicates that those who had even 1 week of on-site practicum acquired greater practical learning in response to nursing problems than those who underwent only on-campus training [9]. Furthermore, surveys targeting newly graduated nurses have revealed that the quantity and quality of clinical practicums during school significantly influence postgraduate practical abilities [10].

A lack of practical competence is not only an educational issue but also a mental health concern. Among different occupational categories in health care, novice nurses report the highest stress levels, with “anxiety over one’s own nursing competence” cited as a major contributing factor [11]. This suggests that the limitations on clinical practicums induced by the COVID-19 pandemic may have a direct impact on the concerned individuals’ mental health.

Of particular concern is burnout, which consists of 3 dimensions: emotional exhaustion, depersonalization, and reduced personal accomplishment [12]. Nurses are regularly exposed to multiple stressors, such as excessive workload, interpersonal conflicts, and shift work, which can easily induce burnout. If left unaddressed, burnout can progress to serious outcomes such as depressive symptoms and increased turnover intentions [13].

Among younger nurses, difficulties adapting to clinical settings and low self-efficacy are known to exacerbate burnout [14]. Newly graduated nurses experience a period of intense emotional, physical, and intellectual stress as they transition from the role of student to that of a professional nurse [15]. This phase is characterized by uncertainty, role confusion, and loss of confidence, all of which can heighten vulnerability to burnout. From a broader perspective, work adaptation theories such as the theory of work adjustment by Dawis and Lofquist [16] emphasize the importance of achieving a balance between individual needs and environmental demands. Insufficient adaptation during this early stage can lead to dissatisfaction, stress, and eventual turnover. Therefore, for nurses whose practicums were affected by the COVID-19 pandemic, mental health support systems in the workplace, as well as educational interventions aimed at improving interpersonal and stress-coping skills, are essential.

Moreover, social support from supervisors and colleagues, the maintenance of work-life balance, and cultivation of psychologically safe work environments are all critical for preventing burnout and maintaining mental health. Nevertheless, research on the impact of changes in clinical practicums during the COVID-19 pandemic on the mental health of newly graduated nurses remains limited. As nurse mental health is closely linked to turnover and the quality of care, more research should focus on the quality of educational experiences during training to inform effective support strategies.

### Purpose of This Study

This study aimed to longitudinally examine how changes in clinical practicums owing to the COVID-19 pandemic affected the mental health of nurses who graduated in the academic year 2021‐2022. We adopted the following terminology related to clinical practicums [17]:

- *Clinical practicum* refers to educational experiences designed to cultivate the ability to apply knowledge and skills in practical nursing settings by integrating and understanding nursing theory and practice. It includes learning one’s role as a member of a health care team and developing the ability to provide nursing care through collaboration and cooperation with professionals in the health, medical, and welfare fields. The practicum encompasses content in adult (chronic and acute care), gerontological, psychiatric, maternal, and pediatric nursing.
- *Integration and practice of nursing* refers to educational experiences aimed at understanding both membership and leadership roles as a nurse in team-based, interprofessional medical care settings. Additionally, it includes developing fundamental abilities for nursing management.

## Methods

### Participants

This study targeted 225 nurses who had completed basic nursing education and were employed for the first time within 1 year after graduation in the academic year 2021-2022 at 39 hospitals (out of the total of 336 hospitals) in Hyogo Prefecture, Japan, who consented to participate.

### Research Design and Survey Periods

We conducted a quantitative longitudinal study over 3 time points: June 2022, September 2022, and December 2022. The first, second, and third surveys were conducted from June 1 to 30, 2022; September 1 to 30, 2022; and December 1 to 31, 2022, respectively.

### Survey Content

Basic demographic attributes assessed included sex, age, years of experience, and department. Regarding the impact of clinical practicums on students, participants subjectively rated their perceived impact of clinical practicums (in adult, gerontological, psychiatric, pediatric, and maternal nursing) and the integration and practice of nursing practicum on a 5-point Likert scale ranging from “very much affected” (5 points) to “not affected at all” (1 point).

The participants were also asked to indicate the actual form of clinical practicum implementation from the following 5 options: “all practicums conducted in clinical settings,” “partially substituted with on-campus training,” “partially substituted with online training,” “all substituted with on-campus training,” and “all substituted with online training.” Subsequently, they rated the degree of difficulty experienced in clinical practice owing to the changes in clinical practicums on a 5-point Likert scale from “very much felt” (5 points) to “not felt at all” (1 point).

### Measurement Tools

#### Stressors

We used the Nursing Job Stressor Scale developed by Higashiguchi et al [18]. This instrument contains 33 items describing potentially stressful situations in nursing and includes the following 7 subscales: “conflict with other nursing staff,” “nursing role conflict,” “conflict with physicians/autonomy,” “dealing with death and dying,” “qualitative workload,” “quantitative workload,” and “conflict with patients.” Higher scores indicate greater perceived stress.

#### Burnout

We measured burnout using the Maslach Burnout Inventory [12] originally developed by Maslach et al [19] and later adapted by Tao and Kubo [20]. The instrument consists of 17 items across 3 dimensions: “emotional exhaustion,” “depersonalization,” and “personal accomplishment.” The reliability and validity of the scale have been confirmed. Responses are provided on a 5-point scale from “always present” to “absent.” Higher scores in “emotional exhaustion” and “depersonalization” and lower scores in “personal accomplishment” indicate a higher likelihood of burnout.

#### Intention to Leave the Job

We assessed participants’ intention to leave using items developed by Ohue et al [13], which were based on the categories defined by Tsuchie and Nakamura [21]. The items used were “wants to quit working as a nurse,” “wants to switch hospitals or departments,” and “wants to continue working as a nurse.” Responses were provided on a 5-point Likert scale ranging from “always present” to “absent,” with higher scores indicating a stronger intention to leave.

#### Data Collection Procedure

We used the paid version of Google Forms to create both the survey and a corresponding QR code. After obtaining approval from the ethics review board, we explained, both in writing and verbally, the purpose and content of the study to the directors of nursing at participating hospitals to obtain their consent. Upon approval, we sent the research materials, including a printed explanation of the study and a survey form with a QR code, to the nursing departments of each hospital. Ward nurse managers were then responsible for explaining the study to eligible nurses and distributing the survey invitation and questionnaire.

Nurses who agreed to participate in the study accessed the survey via the QR code provided on the printed form. Responses to all instruments were collected at the 3 aforementioned time points using the same survey content. The survey was anonymous. The participants completed the first survey via the QR code on the printed form. The second and third surveys were distributed via email to the addresses provided by the participants. We conducted longitudinal data linkage using voluntarily registered email addresses, which were deleted upon completion of the study.

### Data Analysis

We calculated descriptive statistics (frequencies and percentages) for the participants’ demographic characteristics and the mean and SD for their ages. We categorized the participants into 2 groups for each practicum domain: a low-impact group (responses of “not affected at all,” “not very affected,” or “neutral”) and a high-impact group (responses of “somewhat affected” or “strongly affected”) regarding the perceived impact of the clinical practicums and the integration and practice of nursing practicum.

After confirming normality, we conducted an ANOVA to examine differences in mental health variables by survey time (June 2022, September 2022, and December 2022) and practicum impact group (high vs low). We adopted a significance level of *P*<.05 for all analyses. All statistical analyses were performed using SPSS Statistics for Windows (version 26; IBM Corp).

### Ethical Considerations

This study was conducted with the approval of the Ethics Committee of the University of Hyogo. We submitted written requests for participation to the administrators of participating facilities. Only those facilities that agreed were included in the study. We provided a written explanation of the study to all potential participants. Only those who gave informed consent were included. The written explanation clarified that participation or withdrawal from the study was entirely voluntary and would not result in any disadvantage. It also clarified that the data would be used exclusively for this study. All collected data were statistically processed using code numbers, ensuring privacy and confidentiality, and no personally identifiable information was disclosed. Participants were compensated for their time and contribution to this research. Compensation included a gift card valued at JP ¥500 (US $3.19). Compensation was determined to be appropriate, noncoercive, and commensurate with the expected time and effort required for participation. Information about compensation was clearly stated in the consent form and communicated to participants before enrollment.

## Results

### Participant Demographics

Table 1 shows the number of participants and their demographic data. In the first survey, conducted in June 2022, a total of 225 newly graduated nurses responded (n=18, 8% men and n=207, 92% women). The second survey, conducted in September 2022, yielded 140 responses (n=10, 7.1% men and n=130, 92.9% women), and the third survey in December 2022 had 85 respondents (n=5, 5.9% men and n=80, 94.1% women). Of these, 73 nurses (n=2, 2.7% men and n=71, 97.3% women) completed all 3 surveys without missing data and, thus, were included in the final analysis. This represented a response rate of 32.4% (73/225). The average age of the participants was 23.23 (SD 3.68) years. The main reasons for dropping out of the study included changing jobs, changes in work arrangements, and refusal to answer the survey questions. We conducted 2-tailed independent *t* tests to compare completers and noncompleters at baseline for stressors, burnout, and turnover intention scores, finding no significant differences. As such, attrition did not result in substantial systematic bias.

**Table 1.** Basic attributes of the participants (N=73).

Attributes	Participants, n (%)
Sex	
Male	2 (2.7)
Female	71 (97.3)
Education	
Vocational school	19 (26.0)
Junior college	1 (1.4)
University	47 (64.4)
Postgraduate school	6 (8.2)
Qualification	
Nurse	71 (97.3)
Midwife	2 (2.7)
Work shift	
Day shift only	13 (17.8)
3-shift rotation	15 (20.5)
2-shift rotation	45 (61.6)
Department	
General ward	49 (67.1)
Obstetrics and gynecology ward	1 (1.4)
Pediatric ward	5 (6.8)
Intensive care unit	11 (15.1)
Operating room	6 (8.2)
Psychiatric ward	1 (1.4)
Acceptance of patients with COVID-19 at their hospital	
Accepting patients with severe disease	13 (17.8)
Accepting patients with moderate disease	5 (6.8)
Accepting patients with mild disease	27 (37)
Not accepting patients	28 (38.4)

### Perceived Impact of Each Clinical Practicum

Table 2 presents the results on the perceived impact of the integration and practice of nursing practicum. A total of 11% (8/73) of the respondents reported being “not affected at all,” 21.9% (16/73) reported being “not very affected,” 5.5% (4/73) reported being “neutral,” 34.2% (25/73) reported being “somewhat affected,” and 27.4% (20/73) reported being “strongly affected.” Regarding the actual implementation method of this practicum, 30.1% (22/73) of the participants experienced it entirely in clinical settings, 46.6% (34/73) experienced a partial substitution with on-campus training, 5.5% (4/73) experienced a partial substitution with online training, 12.3% (9/73) had full substitution with on-campus training, and 5.5% (4/73) experienced full substitution with online training. Regarding domain-specific clinical practicums (adult, geriatric, psychiatric, pediatric, and maternal nursing), most participants reported being “somewhat affected” (38/73, 52.1%) followed by “strongly affected” (19/73, 26%). Regarding the implementation of domain-specific clinical practicums, most participants (55/73, 75.3%) experienced partial on-campus substitution.

**Table 2.** Degree of impact of each clinical practicum on students (N=73).

	Participants, n (%)
Integrated nursing practicum	
Not affected at all	8 (11)
Not very affected	16 (21.9)
Neutral	4 (5.5)
Somewhat affected	25 (34.2)
Strongly affected	20 (27.4)
Specific impact on the integrated nursing practicum	
Entirely clinical practicum	22 (30.1)
Partial use of on-campus training for clinical practicum	34 (46.6)
Partial use of online training for clinical practicum	4 (5.5)
Entirely on-campus practicum	9 (12.3)
Entirely online practicum	4 (5.5)
Each clinical practicum (adult, maternity, pediatric, geriatric, and psychiatric nursing**)**	
Not affected at all	2 (2.7)
Not very affected	14 (19.2)
Neutral	0 (0)
Somewhat affected	38 (52.1)
Strongly affected	19 (26)
Specific impact on the domain-specific practicum	
Entirely clinical practicum	4 (5.5)
Partial use of on-campus training for clinical practicum	55 (75.3)
Partial use of online training for clinical practicum	9 (12.3)
Entirely on-campus practicum	4 (5.5)
Entirely online practicum	1 (1.4)

### Perceived Practicum Impact and Difficulty in Clinical Practice by Period

To examine the relationship between practicum experience and perceived difficulty in clinical practice, we divided participants into high- and low-impact groups based on their responses. Those who answered with “somewhat affected” or “strongly affected” were categorized into the high-impact group, whereas those who responded with “not affected at all,” “not very affected,” or “neutral” were categorized into the low-impact group. We conducted an ANOVA with time (June 2022, September 2022, and December 2022) and practicum impact level as factors. The analysis revealed significant interaction effects across the 3 time points (Table 3). Further analysis of simple main effects showed that, at all survey time points, the high-impact group for both the integration and domain-specific practicums reported significantly greater difficulty in clinical practice.

**Table 3.** Perceived practicum impact and difficulty in clinical practice by period (time 1: June 2022; time 2: September 2022; time 3: December 2022). Scores represent responses on a 5-point Likert scale.

Time	Low impact for specific clinical practicums on students (score), mean (SD)		High impact for specific clinical practicums on students (score), mean (SD)		Main effect—specific clinical practicums			Main effect—integrated clinical practicum			Interaction		
	Low impact for integrated clinical practicum on students	High impact for integrated clinical practicum on students	Low impact for integrated clinical practicum on students	High impact for integrated clinical practicum	*F* test (*df*)	*P* value	η^2^	*F* test (*df*)	*P* value	η^2^	*F* test (*df*)	*P* value	η^2^
1	2.18 (0.98)	3.50 (0.58)	3.36 (1.15)	3.23 (0.86)	2.00 (1, 59)	.16	0.04	3.46 (1, 59)	.07	0.06	5.08 (1, 59)	.03	0.09
2	3.27 (1.01)	4.00 (0.82)	3.86 (1.23)	2.58 (1.10)	1.28 (1, 59)	.26	0.02	0.56 (1, 59)	.46	0.01	7.33 (1, 59)	.01	0.13
3	3.00 (1.10)	4.00 (0.82)	3.64 (1.22)	2.62 (1.20)	0.90 (1, 59)	.35	0.02	0.00 (1, 59)	.97	0.00	6.70 (1, 59)	.01	0.12

### Perceived Practicum Impact and Stressors by Period

As shown in Table 4, the results of a 2-way ANOVA revealed a significant interaction for “interpersonal stressors in the workplace” in the second survey (September 2022) and “interpersonal stressors with patients” in the third survey (December 2022). In both cases, the high-impact group for the integration practicum reported significantly higher stress levels than the low-impact group for the domain-specific practicums. Although we found no interaction for “stress related to physicians,” we observed significant main effects for both practicum types.

**Table 4.** Perceived practicum impact and stressors by period (time 1: June 2022; time 2: September 2022; time 3: December 2022). Scores represent responses on a 5-point Likert scale.

Time	Low impact for specific clinical practicums on students (score), mean (SD)		High impact for specific clinical practicums on students (score), mean (SD)		Main effect—specific clinical practicums			Main effect—integrated clinical practicum			Interaction		
	Low impact for integrated clinical practicum on students	High impact for integrated clinical practicum on students	Low impact for integrated clinical practicum on students	High impact for integrated clinical practicum on students	*F* test (*df*)	*P* value	η^2^	*F* test (*df*)	*P* value	η^2^	*F* test (*df*)	*P* value	η^2^
The total strain													
1	2.46 (0.25)	2.25 (0.7)	2.48 (0.64)	2.44 (0.71)	0.25 (1, 59)	.62	0	0.37 (1, 59)	.54	0.01	0.17 (1, 59)	.68	0
2	2.45 (0.68)	2.95 (0.37)	2.51 (0.38)	2.55 (0.56)	0.89 (1, 59)	.35	0.02	2.33 (1, 59)	.13	0.04	1.63 (1, 59)	.21	0.03
3	2.64 (0.62)	2.6 (0.69)	2.7 (0.61)	2.74 (0.66)	0.22 (1, 59)	.64	0	0 (1, 59)	>.99	0	0.04 (1, 59)	.85	0
Nursing role conflict													
1	2.65 (0.75)	3 (1.07)	2.54 (0.66)	2.77 (0.64)	0.53 (1, 59)	.47	0.01	1.47 (1, 59)	.23	0.03	0.06 (1, 59)	.80	0
2	2.69 (0.93)	3.1 (0.68)	2.29 (0.88)	2.67 (0.63)	2.62 (1, 59)	.11	0.05	2.35 (1, 59)	.13	0.04	0 (1, 59)	.96	0
3	2.69 (0.81)	2.85 (1.01)	2.29 (1.13)	2.69 (0.78)	0.87 (1, 59)	.35	0.02	0.88	.35	0.02	0.17 (1, 59)	.68	0
Conflict with physicians													
1	2.19 (0.69)	2.14 (1.44)	2.4 (0.98)	2.27 (1.09)	0.23 (1, 59)	.63	0	0.07	.79	0	0.01 (1, 59)	.91	0
2	2.22 (0.89)	2.82 (0.65)	2.76 (0.75)	2.43 (0.72)	0.08 (1, 59)	.78	0	0.29	.59	0.01	3.29 (1, 59)	.08	0.06
3	2.31 (1.05)	2.43 (0.58)	1.98 (1.31)	2.53 (0.84)	0.11 (1, 59)	.74	0	0.98	.33	0.02	0.41 (1, 59)	.52	0.01
Death and dying													
1	1.55 (0.7)	1.3 (0.89)	1.67 (1.36)	1.62 (1.09)	0.37 (1, 59)	.54	0.01	0.16	.69	0	0.07 (1, 59)	.79	0
2	1.95 (1.06)	2.9 (0.42)	1.7 (1.06)	2.01 (1)	2.85 (1, 59)	.10	0.05	3.51	.07	0.06	0.92 (1, 59)	.34	0.02
3	1.82 (1.29)	2.4 (0.63)	1.66 (1.39)	2.06 (1.17)	0.37 (1, 59)	.55	0.01	1.43	.24	0.03	0.05 (1, 59)	.83	0
Qualitative workload													
1	1.73 (1.35)	1.75 (1.37)	1.64 (1.25)	1.55 (1.38)	0.1 (1, 59)	.75	0	0.01	.94	0	0.02 (1, 59)	.90	0
2	1.77 (1.36)	2.56 (0.88)	1.54 (1.16)	1.88 (1.09)	1.39 (1, 59)	.24	0.03	2.15	.15	0.04	0.32 (1, 59)	.57	0.01
3	1.91 (1.31)	1.88 (0.85)	1.43 (1.22)	1.88 (1.15)	0.36 (1, 59)	.55	0.01	0.27	.61	0.01	0.36 (1, 59)	.55	0.01
Quantitative workload													
1	3.25 (0.68)	2.5 (0.26)	3.3 (0.73)	3.07 (0.71)	1.76 (1, 59)	.19	0.03	4.53	.04	0.08	1.28 (1, 59)	.26	0.02
2	3.04 (0.86)	3.1 (0.74)	3.2 (0.68)	2.99 (0.85)	0.01	.92	0	0.07	.79	0	0.25 (1, 59)	.62	0
3	3.35 (0.74)	2.95 (1.1)	2.94 (1.25)	3.07 (0.88)	0.19	.67	0	0.17	.68	0	0.63 (1, 59)	.43	0.01
Conflict with patients													
1	3.25 (0.7)	2.9 (0.2)	3.27 (0.66)	3.19 (0.75)	0.44	.51	0.01	0.86	.36	0.02	0.35 (1, 59)	.56	0.01
2	3.2 (0.93)	3.15 (0.72)	3.31 (0.7)	3.15 (0.9)	0.04	.84	0	0.14	.71	0	0.04 (1, 59)	.85	0
3	3.47 (0.69)	3.15 (1.01)	3.31 (1.09)	3.18 (0.8)	0.05	.82	0	0.61	.44	0.01	0.1 (1, 59)	.75	0
Conflict with other nursing staff													
1	2.73 (1.01)	1.88 (0.85)	2.32 (1.07)	2.52 (0.98)	0.13	.73	0	0.94	.34	0.02	2.43 (1, 59)	.13	0.05
2	2.68 (0.92)	3.13 (0.85)	2.39 (1.24)	2.75 (1.11)	0.81	.37	0.02	1.18	.28	0.02	0.01 (1, 59)	.91	0
3	3.32 (0.56)	2.25 (0.87)	3.36 (0.63)	3.21 (0.9)	3.69	.06	0.07	5.43	.02	0.1	3.14 (1, 59)	.08	0.06

### Perceived Practicum Impact and Burnout by Period

We also examined burnout using a 2-way ANOVA (Table 5). We did not find statistically significant interaction effects but identified significant main effects. In the third survey (December 2022), scores for “emotional exhaustion” were significantly higher in the high-impact groups for both the integration and domain-specific practicum types compared with the corresponding low-impact groups.

**Table 5.** Perceived practicum impact and burnout by period (time 1: June 2022; time 2: September 2022; time 3: December 2022). Scores represent responses on a 5-point Likert scale.

Time	Low impact for specific clinical practicums on students (score), mean (SD)		High impact for specific clinical practicums on students (score), mean (SD)		Main effect—specific clinical practicums			Main effect—integrated clinical practicum			Interaction		
	Low impact for integrated clinical practicum on students	High impact for integrated clinical practicum on students	Low impact for integrated clinical practicum on students	High impact for integrated clinical practicum on students	*F* test (*df*)	*P* value	η^2^	*F* test (*df*)	*P* value	η^2^	*F* test (*df*)	*P* value	η^2^
Emotional exhaustion													
1	3.71 (0.85)	3.8 (0.71)	3.67 (1.06)	3.51 (0.9)	0.28	.60	0.01	0.01	.91	0	0.17	.68	0
2	3.67 (0.76)	3.55 (0.87)	3.93 (0.99)	3.85 (0.91)	0.83	.37	0.02	0.11	.74	0	0	.95	0
3	3.6 (0.69)	3.05 (1.11)	4.13 (0.63)	3.52 (0.97)	3	.09	0.06	4.11	.05	0.07	0.01	.91	0
Depersonalization													
1	2.02 (0.72)	2.08 (0.8)	2.12 (1.29)	1.97 (0.97)	0	.99	0	0.01	.91	0	0.1	.76	0
2	2.36 (0.98)	2.08 (0.7)	2.73 (1.22)	2.45 (0.94)	1.14	.29	0.02	0.67	.42	0.01	0	>.99	0
3	2.32 (0.66)	2.08 (0.65)	2.43 (1.03)	2.35 (0.95)	0.37	.54	0.01	0.27	.61	0.01	0.06	.80	0
Personal accomplishment													
1	7.55 (3.72)	10.5 (4.73)	5.79 (4.08)	7.38 (4.79)	2.7	.11	0.05	2.35	.13	0.04	0.21	.65	0
2	7.09 (3.86)	11.75 (4.65)	6.64 (4.68)	7.15 (5.21)	2.44	.12	0.05	2.57	.12	0.05	1.65	.20	0.03
3	5.73 (3.85)	8.25 (3.3)	8.71 (6.51)	6.42 (3.9)	0.14	.71	0	0.01	.94	0	2.36	.13	0.04

### Perceived Practicum Impact and Intention to Leave by Period

Finally, regarding intention to leave the job, we observed a significant interaction effect in the second survey (September 2022) for the item “want to continue working as a nurse” (Table 6). Simple main effect analysis indicated that, in the low-impact group for the integration practicum, those in the low-impact group for the domain-specific practicums showed a significantly stronger intention to remain in the profession than those in the high-impact group. We found no interaction effect for the item “want to quit being a nurse” but noted a significant main effect in the third survey (December 2022), with the high-impact group for the domain-specific practicums showing a significantly greater intention to leave the nursing profession compared with the low-impact group.

**Table 6.** Perceived practicum impact and intention to leave by period (time 1: June 2022; time 2: September 2022; time 3: December 2022). Scores represent responses on a 5-point Likert scale.

Time	Low impact for specific clinical practicums on students (score), mean (SD)		High impact for specific clinical practicums on students (score), mean (SD)		Main effect—specific clinical practicums			Main effect—integrated clinical practicum			Interaction		
	Low impact for integrated clinical practicum on students	High impact for integrated clinical practicum on students	Low impact for integrated clinical practicum on students	High impact for integrated clinical practicum on students	*F* test (*df*)	*P* value	η^2^	*F* test (*df*)	*P* value	η^2^	*F* test (*df*)	*P* value	η^2^
Wants to quit working as a nurse													
1	2.64 (1.43)	2.75 (0.96)	2.86 (1.41)	2.5 (1.21)	0	.97	0	0.08	.78	0	0.29	.59	0.01
2	2.27 (1.19)	2.75 (1.71)	2.36 (1.39)	1.92 (1.29)	0.69	.41	0.01	0	.96	0	1.04	.31	0.02
3	3.55 (1.21)	2.5 (1)	3.71 (1.14)	3.62 (1.02)	3.07	.09	0.06	2.44	.12	0.05	1.67	.20	0.03
Wants to switch hospitals													
1	3.09 (1.58)	2.75 (0.5)	3.5 (1.09)	3.08 (1.32)	0.72	.40	0.01	0.78	.38	0.02	0.01	.92	0
2	3 (1.41)	2.25 (1.89)	2.64 (1.6)	2.54 (1.3)	0.01	.94	0	0.78	.38	0.01	0.44	.51	0.01
3	3.55 (1.21)	3.5 (1.73)	2.71 (1.2)	3.31 (1.32)	1.38	.25	0.03	0.39	.53	0.01	0.54	.47	0.01
Wants to continue working as a nurse													
1	2.82 (1.17)	2.5 (1.29)	3.14 (1.29)	3.12 (1.37)	1.15	.29	0.02	0.16	.70	0	0.11	.74	0
2	2.27 (1.01)	3.25 (1.26)	3 (1.57)	2.31 (1.44)	0.05	.82	0	0.09	.76	0	3.2	.08	0.06
3	2.91 (1.22)	3.5 (0.58)	3.43 (1.45)	3.27 (1.43)	0.1	.75	0	0.22	.64	0	0.67	.42	0.01

## Discussion

### Principal Findings

This longitudinal study examined the impact of COVID-19 pandemic–induced changes in clinical practicums on newly graduated nurses’ perceived difficulty in clinical practice, stressors, burnout, and intention to leave the profession using a 2-way ANOVA by response periods. We analyzed the extent of the perceived impact of clinical practicums. Nearly 62% of the participants (45/73) reported effects on the integration and practice of nursing practicum, whereas 78% (57/73) reported effects on domain-specific practicums. The most common form of alteration was partial substitution with on-campus training. These results are consistent with those reported by Sugawara et al [5] that most newly graduated nurses in this cohort experienced changes in their practicums owing to the COVID-19 pandemic. In our study, participants who experienced full clinical training demonstrated higher practical problem-solving ability than those whose training was replaced with other formats. Given that the 2022 cohort experienced shortened practicums or substitutions with online or on-campus alternatives, these modifications may have influenced their clinical readiness and problem-solving behaviors.

Regarding perceived difficulty in clinical practice, we found significant interaction effects at the 3 time points (June 2022, September 2022, and December 2022) between the perceived impact of domain-specific and integration practicums. Our analysis of simple main effects revealed that participants who experienced greater impacts from both practicum types reported significantly higher difficulty in clinical practice. Taguchi et al [22] previously reported that newly graduated nurses during the COVID-19 pandemic experienced challenges in comprehensively assessing patients’ needs across physical, psychological, and social domains, suggesting that reduced practicum time may have hindered the acquisition of these skills. Taken together, these findings indicate that the restriction on clinical learning opportunities during the COVID-19 pandemic substantially impeded the development of practical skills, manifesting as increased perceived difficulty in clinical settings.

In terms of stressors, we identified significant interactions for “interpersonal strain within the workplace” in September 2022 and “interpersonal strain with patients” in December 2022. In both cases, those who experienced higher impacts from the integration practicum reported significantly higher levels of strain than those in the low-impact group for the domain-specific practicums. This suggests that reduced practicum opportunities may have compromised the development of interpersonal skills and the ability to adapt to workplace environments.

Although the interaction was not significant for “strain in relationships with physicians,” both practicum types showed significant main effects, indicating their independent contributions to increased stress. In response to the pandemic, institutions revised educational programs to include alternative learning methods, such as observing senior nurses and providing mental health support [23]. However, the lack of direct exposure to interprofessional interactions during practicums may have limited participants’ understanding of nurse-physician relationships, contributing to stress. Alternatively, as Ohue et al [10] suggested, decreased clinical experience may lower self-confidence in nursing practice, which in turn may increase stress related to relationships with physicians. These findings suggest that limitations in practicum opportunities owing to the COVID-19 pandemic may have hindered the acquisition of interpersonal and interprofessional collaboration skills, leading to increased strain in relationships within the workplace and with patients and physicians. Such stressors may ultimately increase the risk of job turnover, highlighting the urgent need for comprehensive support systems in both educational and clinical settings.

Regarding burnout, main effects were evident for “emotional exhaustion” in the December 2022 survey, with higher scores observed in the high-impact groups for both the domain-specific and integration practicum types. This suggests that restrictions in clinical practicum environments contribute to psychological burden, particularly emotional fatigue, after entering clinical practice. Emotional exhaustion is defined as a state of feeling emotionally overextended and depleted by one’s work [24]. During the COVID-19 pandemic, the lack of opportunities to develop interpersonal skills through hands-on training likely rendered newly graduated nurses more vulnerable to early stress and burnout. Manabe et al [23] identified that first-year nurses who recognized stressors related to “lack of nursing competence” also reported lower personal accomplishment, emphasizing the need for support tailored to changes in stress and coping patterns during the first year, as well as the need to enhance nursing competence.

These results can also be interpreted through theoretical frameworks of role transition and work adjustment. Duchscher [15] described the “transition shock” experienced by newly graduated nurses in the acute stage of adaptation, characterized by emotional disorientation, self-doubt, and stress as they transition from student to professional roles. Our findings—higher difficulty in clinical practice, interpersonal strain, and emotional exhaustion among those with reduced practicum experience—are consistent with this concept, suggesting that insufficient practical exposure intensified transition shock during the early employment period. Furthermore, from the perspective of work adaptation theory [16], inadequate preparation during the educational stage may have disrupted the balance between individual abilities and workplace demands, thereby hindering successful adjustment to the professional environment. This mismatch likely contributed to the sustained stress and burnout and increased turnover intention observed in the high-impact groups. Integrating these theoretical perspectives helps explain how educational disruptions translate into psychological maladaptation and occupational instability among new nurses.

Regarding intention to leave, we identified a significant interaction for the item “want to continue working as a nurse” in the September 2022 survey. Simple main effects indicated that, among those in the low-impact group for the integration practicum, those in the low-impact group for domain-specific practicums were significantly more likely to intend to continue in the profession. This suggests that limitations in both practicum types adversely influence the intention to continue in the nursing profession. Meanwhile, we found no interaction for the item “want to quit nursing” but noted a significant main effect in the December 2022 survey, with higher scores among participants who had experienced greater impacts in domain-specific practicums. Ohue et al [13] also reported associations among stressors, burnout, and intention to leave among nurses. In other words, COVID-19 pandemic–related limitations in clinical practicums may contribute to elevated stress and burnout, which then increase turnover intention.

In summary, our study revealed that changes in domain-specific and integration practicums owing to the COVID-19 pandemic significantly impacted not only the practical competencies of newly graduated nurses but also their mental health and career continuity. The participants in the high-impact groups showed elevated difficulty in clinical practice, greater stress, higher emotional exhaustion, and a stronger intention to leave, all of which are potential risk factors for early attrition. Considering the theoretical frameworks of transition shock and work adaptation, these findings highlight the importance of structured transition programs, mentorship, and organizational support to mitigate new nurses’ initial adjustment difficulties and facilitate smoother professional adaptation.

One limitation of our study pertains to the retention rate across the 3 survey time points. Although we confirmed that attrition did not introduce substantial systematic bias, we cannot rule out the possibility that the participants who remained in the study differed in unmeasured ways from those who dropped out. As such, the findings may primarily reflect the experiences of nurses who were able to continue in the profession during the first year. Generalizations to all newly graduated nurses should be made with caution.

Moving forward, follow-up systems, mental health support, and continuing education to strengthen clinical skills are essential for graduates whose training was adversely impacted because of the pandemic. Future pandemics or emerging infectious diseases may again prevent clinical training in actual health care settings. Therefore, strategies for ensuring meaningful clinical practicums even under restrictive conditions must be explored. Future practicum models should be strengthened and specified to better prepare nursing students for clinical realities. For example, hybrid models combining on-campus simulation with limited in-person clinical exposure could allow students to practice technical and interpersonal skills safely. Structured mentorship programs, where experienced nurses guide students through critical learning points even under restricted conditions, may enhance practical competence and confidence. Additionally, short-term, intensive “juvenile monetary practicums” or condensed clinical rotations focused on high-priority skills could be integrated to ensure essential hands-on experience without compromising safety. Implementing these concrete strategies may mitigate the negative impact of restricted clinical access and support new nurses’ smoother transitions into professional practice.

### Conclusions

This study examined the impact of COVID-19–related changes in clinical training on newly graduated nurses’ perceived difficulty in clinical practice, stressors, burnout, and intention to leave across 3 survey time points. The findings revealed that newly graduated nurses who subjectively perceived a greater impact from both domain-specific and integration practicum types reported higher levels of difficulty in clinical practice and stressors, with the influence of the integration practicum being particularly pronounced. In the second survey (September 2022), the intention to continue working as a nurse was significantly higher among those who perceived less impact from the integration practicum, indicating that practicum experiences may impact career commitment. In the third survey (December 2022), emotional exhaustion was significantly higher in the high-impact group, suggesting a relationship with burnout.

Our results suggest that the quality and extent of clinical practicum experiences significantly influence the psychological burden and occupational retention intentions of newly graduated nurses. For those whose training was adversely impacted because of the COVID-19 pandemic, continuous support systems and enhanced mental health care in clinical settings are essential.
